# Long non-coding RNAs in cutaneous biology and keratinocyte carcinomas

**DOI:** 10.1007/s00018-020-03554-3

**Published:** 2020-05-27

**Authors:** Minna Piipponen, Liisa Nissinen, Veli-Matti Kähäri

**Affiliations:** 1 Department of Dermatology, University of Turku and Turku University Hospital, Hämeentie 11 TE6, 20520 Turku, Finland; 2 Cancer Research Laboratory, Western Cancer Centre of the Cancer Center Finland (FICAN West), University of Turku and Turku University Hospital, Turku, Finland

**Keywords:** Skin cancer, Basal cell carcinoma, Cutaneous squamous cell carcinoma, Epidermis, Wound repair, Ultraviolet radiation

## Abstract

Long non-coding RNAs (lncRNAs) are a largely uncharacterized group of non-coding RNAs with diverse regulatory roles in various biological processes. Recent observations have elucidated the functional roles of lncRNAs in cutaneous biology, *e.g.* in proliferation and differentiation of epidermal keratinocytes and in cutaneous wound repair. Furthermore, the role of lncRNAs in keratinocyte-derived skin cancers is emerging, especially in cutaneous squamous cell carcinoma (cSCC), which presents a significant burden to health care services worldwide and causes high mortality as metastatic disease. Elucidation of the functions of keratinocyte-specific lncRNAs will improve understanding of the molecular pathogenesis of epidermal disorders and skin cancers and can be exploited in development of new diagnostic and therapeutic applications for keratinocyte carcinomas. In this review, we summarize the current evidence of functionally important lncRNAs in cutaneous biology and in keratinocyte carcinomas.

## Introduction

Skin cancers are the most common cancer types globally with increasing incidence [1, 2]. Melanoma, basal cell carcinoma (BCC), and cutaneous squamous cell carcinoma (cSCC) are the three major types of skin cancer. Cumulative exposure to ultraviolet radiation (UVR) is a common risk factor for skin cancers, but they differ with respect to mutational profiles and alterations in cellular signaling pathways [3]. Melanoma originates from melanocytes, whereas BCC and cSCC originate from epidermal keratinocytes and are, therefore, called keratinocyte carcinomas (KC). The best preventive measure against skin cancer is avoiding excessive and cumulative exposure to sunlight and other sources of UVR. In addition, early detection and treatment is pivotal for the prognosis of the disease. The mortality rates for skin cancers vary between populations. However, taking into account the considerably higher incidence of KC over melanoma, it is estimated that the global mortality rate for all non-melanoma skin cancers (NMSCs) including BCC and cSCC, is even higher than for melanoma [4].

A significant proportion of human genome encodes non-coding RNAs (ncRNAs), including ribosomal RNA (rRNA) and transfer RNA (tRNA), and other functionally relevant ncRNAs, roughly categorized to small (sncRNAs) and long non-coding RNAs (lncRNAs) [5]. MicroRNAs (miRNAs) present an evolutionary conserved subgroup of sncRNAs deregulated in different cancers, including BCC and cSCC [6, 7]. LncRNAs are single-stranded RNA molecules larger than 200 nucleotides in size, lacking protein-coding capacity and sequence conservation [8]. It has become increasingly evident that they regulate a variety of cellular functions, and that aberrant expression of lncRNAs plays a role in various pathological conditions including cancer [9].

The mutational background for cSCC and BCC is well documented, and several driver mutations in protein coding genes have been identified [10–17]. These same driver gene mutations are also found frequently in epidermal keratinocytes in normal sun-exposed skin [18], indicating, that also other factors, *e.g.* changes in non-coding genes and the microenvironment, are also necessary for development of cSCC [19]. Mutations in the non-coding regions of genome can affect chromatin structure, transcription factor binding, and gene expression [20]. Moreover, these mutations may alter expression or secondary structure of lncRNAs or interfere with lncRNA interaction with other regulatory factors [21]. The consequence of non-coding mutations in lncRNA expression and function in cutaneous carcinogenesis and skin cancer development is largely unknown. However, recent evidence suggests that lncRNAs participate in the complex cancer signaling network in skin malignancies. Elucidation of their role in cutaneous biology is likely to reveal new molecular targets for diagnostics and therapeutic intervention. In this review, we summarize the current findings of the function of lncRNAs in cutaneous biology and in keratinocyte carcinomas.

## Keratinocyte carcinomas

Keratinocyte carcinomas BCC and cSCC are the most common forms of skin cancer with increasing incidence globally [22, 23]. The primary cause for KCs is chronic exposure to UVR, and other important risk factors include immunosuppression, human papillomavirus infection, and chronic cutaneous ulceration [22–24]. While BCC is the most common human malignancy, cSCC accounts for the majority of deaths among KCs [25, 26]. In addition, a personal history of KCs is associated with a risk for other cancers [27]. In contrast to BCC, which rarely metastasizes, the risk of metastasis for cSCC is estimated as 1–4% and the prognosis of metastatic cSCC is poor [26]. Overall, the high prevalence of KCs poses a marked burden on health care worldwide and has a major impact on the patients’ quality of life [28].

Development of KC involves accumulation of several molecular and cellular changes. Both BCC and cSCC harbor a substantial mutational burden, mainly due to cumulative UV exposure typically observed as C → T transitions in the DNA [12–19]. Several studies using BCC and cSCC murine models suggest that these cancers arise from multiple cellular origins, *e.g.* from different stem cell populations in the basal layer of the epidermis, hair follicle bulge or sebaceous gland [29].

Despite a high frequency of UV-induced mutations, BCCs and cSCCs do not harbor many common genetic alterations, except inactivation of tumor suppressor p53 [12–19]. Several driver gene mutations have been identified for cSCC, resulting in constitutive activation of HRAS and inactivation of tumor suppressors p53 and NOTCH1 [12–16]. Conversely, BCC is strongly associated with aberrant activation of the Hedgehog signaling pathway due to loss of PTCH1 receptor function and activation of the G protein-coupled receptor SMO [17–19]. Like many other cancers, cSCCs and BCCs are associated with epigenetic deregulation and aberrant DNA methylation, which also contribute to cancer progression [30–34].

Actinic keratoses (AKs) are early precursors of cSCC and Bowen’s disease is in situ cSCC (cSCCIS), where atypical keratinocytes extend throughout the epidermis [35]. If left untreated, these lesions develop to invasive cSCCs. In general, patients with BCCs or resectable primary cSCCs have a good prognosis, whereas metastatic cSCC is associated with poor outcome [26]. Radiation and chemotherapy can be used for advanced and recurrent high-risk tumors that cannot be excised, especially those located in the facial area [36]. Recently, targeted therapies have been approved for therapy of advanced BCC and cSCC. Vismodegib, an inhibitor of Hedgehog pathway, is available for treatment of locally advanced BCC [37]. Immune checkpoint inhibitor, programmed cell death protein-1 (PD-1) blocking monoclonal antibody cemiplimab, has been approved for treatment of patients with locally advanced or metastatic cSCC [38]. Nevertheless, there is an urgent need for additional targeted therapies for advanced cSCCs and for prognostic biomarkers for predicting the risk of recurrence and metastatic potential of cSCC.

## Long non-coding RNAs

Long non-coding RNAs (lncRNAs) are single-stranded RNAs mainly transcribed by RNA polymerase II, which undergo post-transcriptional processing, such as 5′-capping, splicing and polyadenylation [8]. This way lncRNAs closely resemble messenger RNAs (mRNA), but they are not translated to proteins. Some lncRNAs are rapidly degraded after transcription, whereas others are extremely stable [39, 40]. A rapid turnover of lncRNAs enables a dynamic cellular response via specifically induced lncRNAs, for instance in DNA damage, immune response, and cellular differentiation [41–43].

LncRNAs are poorly conserved between species [44–46]. In general, lncRNAs are considered larger than 200 nucleotides in size. This division, however, is not strict, as some lncRNAs are less than 200 nucleotides in size, and some lncRNAs can function both as regulatory lncRNAs and can be processed to sncRNAs [47]. Classification of lncRNAs into distinct subgroups is commonly based on their genomic location.

Long intergenic or intervening non-coding RNAs (lincRNAs) are transcribed from distinct loci, often from their own promoters, whereas intronic lncRNAs are transcribed from intronic regions within protein-coding gene [45, 47]. Sense lncRNAs are transcribed from the sense strand also containing exons of protein-coding genes [47]. Natural antisense transcripts (NATs) are transcribed from the antisense strand of a protein-coding gene, overlapping either exonic or intronic regions [47]. Bidirectional lncRNAs are produced divergently from the same promoter of a protein-coding gene. Circular RNAs (circRNA) are a recently discovered group of lncRNAs structurally different from most lncRNAs. They are produced by back splicing of precursor mRNAs or lncRNAs, resulting in covalently closed circular RNAs without polyadenylation, and they can originate from intronic or exonic transcripts [48].

LncRNAs are specifically expressed during normal physiological processes including cell differentiation and tissue development, whereas untimely and aberrant expression of lncRNAs in various pathological conditions is becoming evident [9–11]. Thus, alternative classification has been suggested, for instance by grouping them into lncRNAs regulating gene expression locally (*cis*) or in distance (*trans*) [49], or by other criteria such as subcellular localization, association with DNA-elements, or functional mechanism [50].

## Molecular functions of lncRNAs

**Fig. 1:**
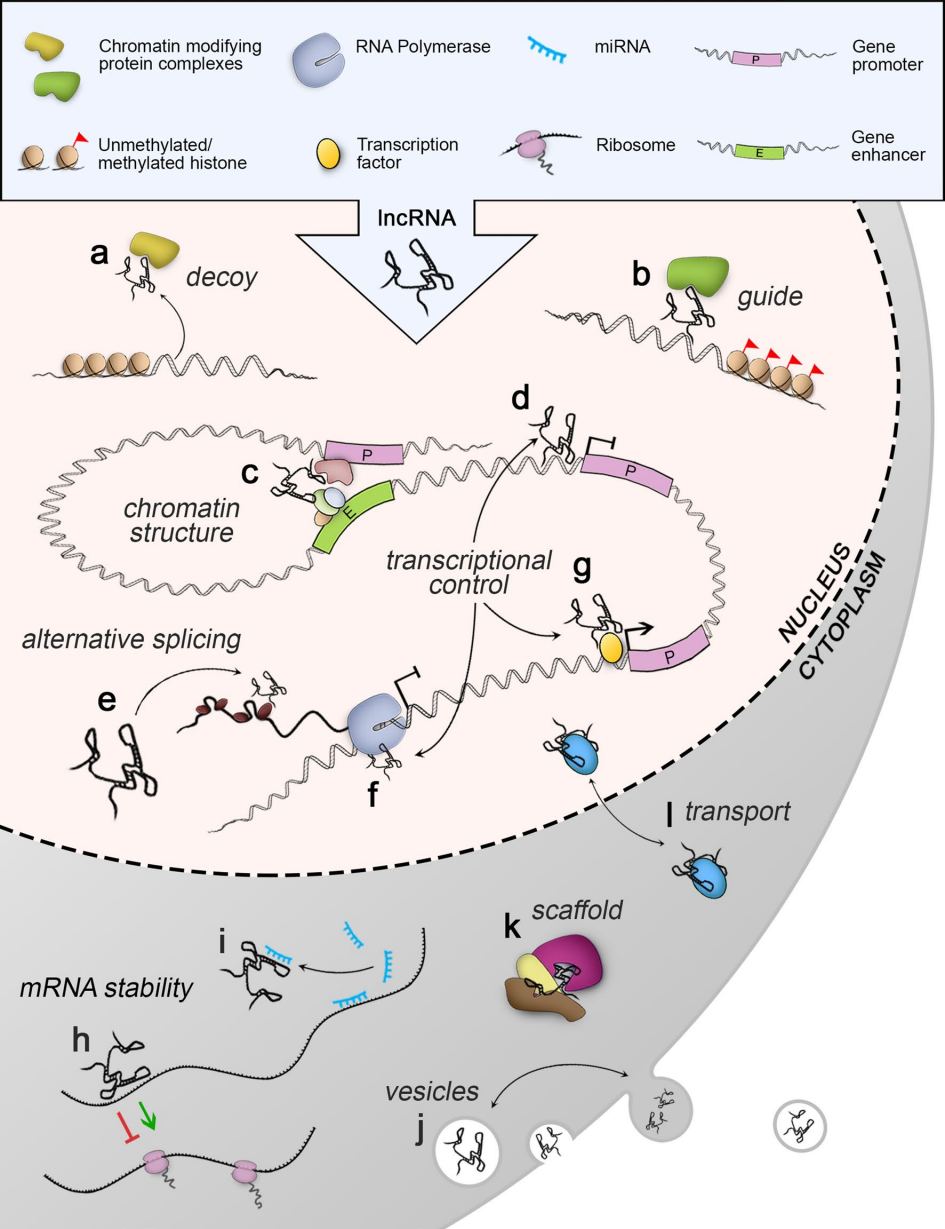
Molecular functions of lncRNAs. Nuclear lncRNAs can regulate epigenetic changes by **a** decoying or **b** guiding chromatin-modifying complexes to specific genomic loci. **c** lncRNAs can induce chromosomal looping to control gene expression by simultaneously binding to protein complexes or specific DNA elements. LncRNAs can inhibit gene transcription **d** by blocking a transcription factor binding site or **f** by binding to RNA polymerase. **g** LncRNAs may contribute to transcriptional activation by guiding transcription factors or other co-factors to gene promoters. **e** LncRNAs can regulate alternative splicing that can occur by lncRNA binding to mRNA and blocking the splice-site. LncRNAs can also recruit and guide splicing factors to the sites of transcription. Cytoplasmic lncRNAs can regulate mRNA stability **h** directly by binding to mRNAs or **i** indirectly by sequestering miRNAs by complementary base pairing. **j** Some lncRNAs can be secreted to extracellular vesicles or exosomes allowing them to mediate intercellular signaling. **k** LncRNAs can serve as scaffolds to promote the assembly of active ribonucleoprotein complexes in the cytoplasm or nucleus. **l** LncRNAs can aid intracellular translocation of proteins

In general, the regulatory role of lncRNAs is based on binding to specific effector molecules by sequence complementarity or structural recognition to mediate gene expression. The single-stranded structure of lncRNAs and folding into unique secondary and tertiary structures gives them the ability to bind to RNA, DNA or proteins and this way control diverse cellular functions [8, 51, 52] (Fig. 1). LncRNAs typically exhibit a strict cell and tissue-specific expression and subcellular localization, indicating strictly controlled regulatory role for distinct lncRNAs [44, 53]. Specific localization of distinct lncRNAs to cytoplasm, nucleus, or other cellular compartments is likely to reflect their function (Fig. 1). In addition, some lncRNAs are secreted in extracellular vesicles and exosomes, and can exert their effect in adjacent cells and in cells in other tissues [54, 55]. In general, lncRNA mechanism of action can be divided into four main types: *signals*, *guides*, *decoys*, and *scaffolds* [51]. Simply, they can also be classified as nuclear lncRNAs in mediating gene transcription [56] or cytoplasmic lncRNAs controlling post-transcriptional events and mRNA stability [57] (Fig. 1).

## LncRNAs in cutaneous biology

### Regulation of epidermal differentiation by lncRNAs

The skin serves as a protective barrier against several environmental threats, including microbes, chemicals, and physical insults, and it also controls water loss and thermoregulation. Skin consists of several different cell types and stem cell populations, which co-operate to maintain and regenerate its structure and function [58]. The epidermal layer of skin is under continuous turnover, as the cells generated from the basal keratinocytes lose their proliferative capacity, commit to terminal differentiation, and move towards skin surface [59]. During this process, keratinocytes undergo major morphological and mechanical changes due to spatiotemporal alteration in their transcriptional program [60]. Several markers for keratinocyte differentiation have been identified and the chromatin dynamics play a crucial role during this process [61–63].

**Fig. 2:**
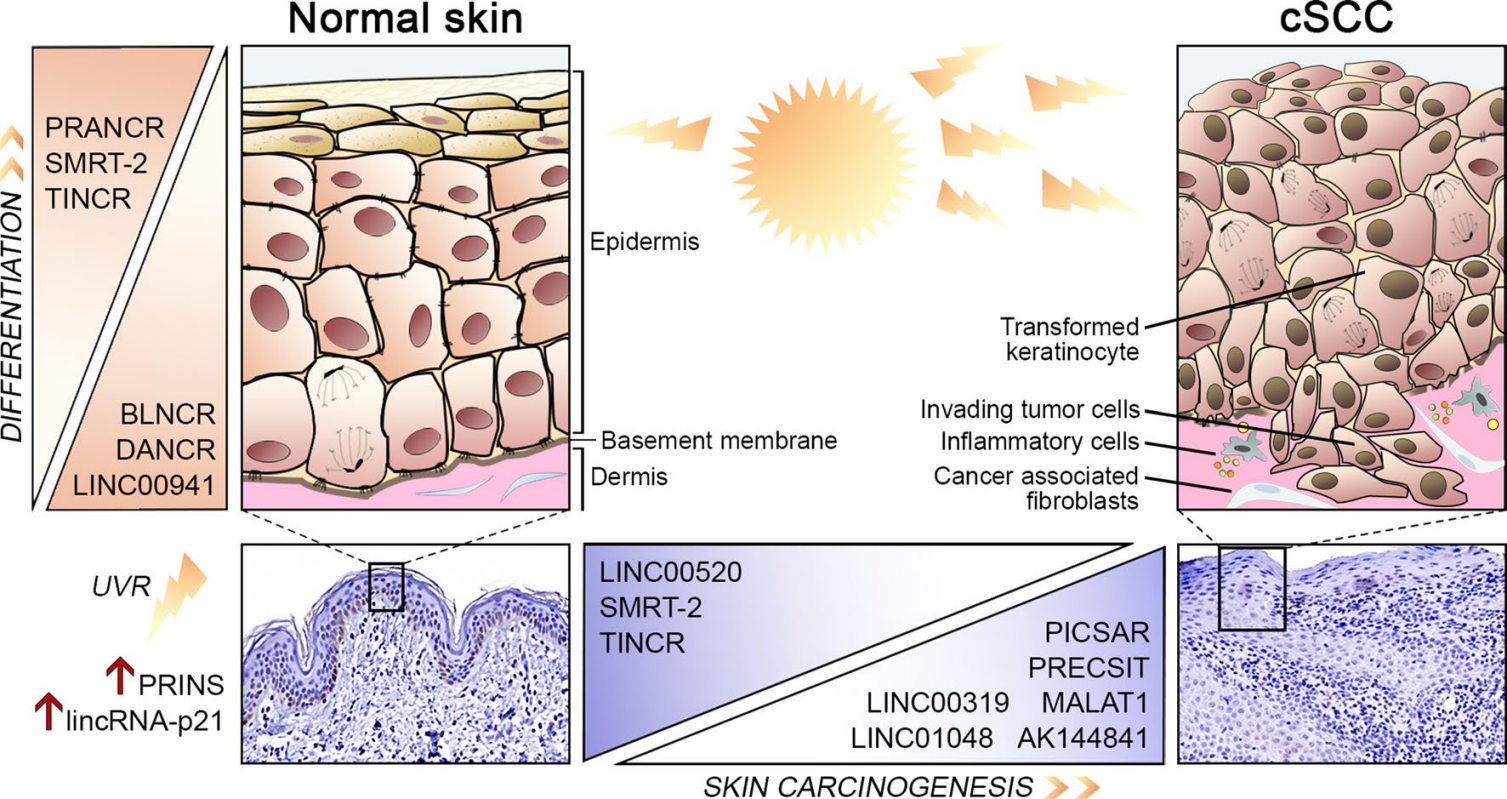
An overview of lncRNAs implicated in epidermal homeostasis in normal skin and in cSCC progression. Solar ultraviolet radiation (UVR) induces a stress response and altered expression of specific lncRNAs, such as *PRINS* and *lincRNA-p21* in normal keratinocytes. Cumulative exposure to UVR predisposes epidermal keratinocytes to DNA damage and malignant transformation, which eventually lead to development of invasive cSCC. Several lncRNAs have been shown to be involved in cutaneous homeostasis. *TINCR*, *PRANCR*, and *SMRT-2* promote and *DANCR*, *BLNCR*, and *LINC00941* inhibit keratinocyte differentiation. Deregulation of lncRNAs is becoming evident in cSCC progression. The expression of *TINCR*, *SMRT-2*, and *LINC00520* is downregulated and the expression of *PICSAR*, *PRECSIT*, *MALAT1*, *AK144841*, *LINC00319*, and *LINC01048* is upregulated in cSCC. These lncRNAs could be used as new prognostic markers and as novel therapeutic targets in cSCC

Transcriptional changes during differentiation of epidermal keratinocytes also involve alterations in the expression of non-coding RNAs and accordingly specific lncRNAs have been implicated in keratinocyte differentiation [64, 65] (Fig. 2). Differentiation antagonizing non-protein coding RNA (*DANCR*) is downregulated during terminal differentiation of keratinocytes and it is required for maintaining the undifferentiated phenotype of epidermal progenitor cells [66]. *DANCR* is a negative regulator of MAF and MAFB transcription factors, which are important regulators of differentiation in various cell types [67]. *DANCR* represses the expression of MAF and MAFB epigenetically by guiding a chromatin-modifying protein complex to the promoters of their genes [66]. In contrast to *DANCR*, terminal differentiation-induced ncRNA (*TINCR*) is highly expressed in differentiating keratinocytes specifically in the suprabasal layers of human epidermis [68]. *TINCR* promotes keratinocyte differentiation by stabilizing mRNAs coding for proteins involved in keratinocyte differentiation *e.g.* transcription factors MAF and MAFB, together with an RNA-binding protein STAU1 [68]. Together, *DANCR* and *TINCR* are able to regulate the expression of a broad range of genes in keratinocytes and this way function as pivotal regulators of epidermal differentiation.

The expression of *LINC00941* is downregulated upon keratinocyte differentiation, and it antagonizes the function of small proline rich protein 5 (SPRR5), which promotes differentiation of keratinocytes [69]. The expression of beta1-adjacent long non-coding RNA (*BLNCR*) is also downregulated during keratinocyte differentiation, preceding downregulation of *ITGB1*, which codes for integrin β1, an epidermal stem cell marker adjacent to *BLNCR* gene [70, 71]. *BLNCR* and *ITGB1* are both transcriptionally regulated by transcription factors p63 and AP-1, and loss of *BLNCR* and *ITGB1* expression may be an early event resulting in loss of proliferative capacity of keratinocytes and in subsequent terminal differentiation [71].

Progenitor renewal associated non-coding RNA, (*PRANCR*), is one of the most recently characterized lncRNAs involved in epidermal homeostasis [72]. Depletion of *PRANCR* leads to reduced proliferative capacity and differentiation of keratinocytes. *PRANCR* regulates the expression of several genes coding for cell cycle regulators, including E2F transcription factor target genes [72]. In addition, H19 imprinted maternally expressed transcript (*H19*) and SCC misregulated transcript-2 (*SMRT-2*) are recently identified lncRNAs induced during differentiation of epidermal keratinocytes [73, 74]. Depletion of *SMRT-2* results in repression of several genes associated with epidermal differentiation and development [74]. These genes are also regulated by zinc finger protein 750 (ZNF750) and Kruppel like factor 4 (KLF4), suggesting that *SMRT-2* functions upstream of the ZNF750-KLF4-axis [74]. ZNF750 functions downstream of p63 in driving epidermal differentiation by upregulating KLF4 [75]. Moreover, ZNF750 upregulates expression of lncRNA *TINCR* [76]. Taken together, these observations provide a regulatory link between *SMRT-2*, ZNF750 and *TINCR* in regulation of epidermal keratinocyte differentiation.

As aberrant keratinocyte differentiation and stem-cell characteristics are involved in KC tumor development [77, 78], it is not surprising that the expression of keratinocyte differentiation inducing lncRNAs, *SMRT-2* and *TINCR*, are strongly downregulated in cSCC [66, 74, 79]. Many of the lncRNAs associated with epidermal differentiation listed here are not implicated in cSCC and it will be important to investigate their mechanistic role in progression of cutaneous cancers.

### LncRNAs in cutaneous wound repair

Cutaneous wound repair is a complex and strictly controlled process, which involves co-operation of several different cell types, including keratinocytes, fibroblasts, endothelial cells, and inflammatory cells [80]. Delayed wound healing resulting in chronic ulcers is usually associated with an underlying condition, such as insufficient arterial or venous circulation, diabetes or prolonged inflammation [80]. Chronic wounds also carry a risk of developing to cSCC [81].

The role of lncRNAs in normal wound repair or in pathogenesis of chronic ulcers is largely unknown. LncRNA expression profile in Marjolin ulcer, a rare, aggressive type of cSCC that evolves in scars or chronic wounds has been reported, but functional characterization of the lncRNAs in this condition is lacking [82]. Growth arrest-specific 5 lncRNA (*GAS5*) is a repressor of glucocorticoid receptor expression, which serves as a tumor suppressor in many cancers [83]. *GAS5* has been shown to promote wound healing by inducing epithelialization and angiogenesis via c-myc inhibition [84]. Metastasis associated lung adenocarcinoma transcript 1 (*MALAT1*), a tumor-promoting lncRNA in many cancers [85] has been shown to stimulate repair of ischemic wounds by promoting migration of human dermal fibroblasts through hypoxia-inducible factor-1α (HIF-1α) signaling [86, 87]. In addition, lncRNA *H19* has been shown to promote wound healing via HIF-1α pathway [88, 89].

Wound and keratinocyte migration-associated lncRNA 1 and 2 (*WAKMAR1* and *WAKMAR2*) are two recently identified lncRNAs, which play an important role in cutaneous wound repair [90, 91]. Expression of *WAKMAR1* is highly upregulated in keratinocytes during wound repair and its expression stimulates keratinocyte migration and wound re-epithelization [90]. Expression of both *WAKMAR1* and *WAKMAR2* is induced by TGF-β and downregulation of their expression inhibits migration of keratinocytes, resulting in delayed wound re-epithelization [90, 91]. Accordingly, the expression of both *WAKMAR1* and *WAKMAR2* is reduced in keratinocytes in the edge of chronic wounds in vivo [90, 91]. Moreover, upregulation of *WAKMAR2* expression inhibits production of inflammatory chemokines by keratinocytes, and this way promotes wound healing [91]. *WAKMAR1* exerts its function by sequestering DNA methyltransferases, resulting in upregulation of the expression of E2F1 transcription factor and subsequent regulation of the expression of its target genes [90].

### Regulation of lncRNAs by UVR

Exposure of skin to UVR induces several cellular responses. Activation of the inflammatory response manifesting as erythema in skin is an acute response after UV exposure [92]. UVR induces DNA damage in epidermal keratinocytes, which triggers a stress response, activation of p53 and DNA repair [93]. UV-induced DNA damage leads to systemic immunosuppression [94–96] which is exploited in treatment of inflammatory skin diseases, such as psoriasis and atopic dermatitis [97].

UVR leads to altered lncRNA expression in epidermal keratinocytes [98], melanocytes, [99] and dermal fibroblasts in culture [100–103]. The biological response of skin to UVA and UVB is distinct due to their different penetration to the skin and they trigger distinct expression pattern of lncRNAs in HDFs [101]. It is possible, that some of these lncRNAs play a role in the early cellular stress response or acute inflammation following exposure to UV. Also, several UV-regulated lncRNAs in keratinocytes show a similar expression trend in cSCC and BCC, suggesting a role for them in epidermal carcinogenesis [98].

A subset of UV-induced lncRNAs has been functionally characterized [102–105]. In keratinocytes, the expression of *lincRNA-p21* is markedly induced by UVB through a p53-dependent mechanism and it exerts a tumor suppressive role by triggering UVB-induced apoptosis and cell cycle arrest [105]. Accordingly, a tumor suppressive function for *lincRNA-p21* has been reported in head and neck SCC [106]. Psoriasis susceptibility-related RNA gene induced by stress (*PRINS*) is a lncRNA induced by UVB and other stress signals, such as serum starvation or translational inhibition in HaCaT cells, an epidermal keratinocyte derived cell line, which lacks functional p53 [104]. Elevated expression of *PRINS* in psoriatic epidermis has also been reported, suggesting a role for *PRINS* in pathogenesis of psoriasis [104].

Vitamin D is photochemically synthesized in the skin by UVB and recent findings support a cancer protecting role for vitamin D [107]. Interestingly, keratinocytes lacking vitamin D receptor show a distinct lncRNA expression pattern with increased expression of oncogenic lncRNAs and decreased expression of tumor-suppressive lncRNAs, including *lincRNA-p21* [108]. It appears, that UVR plays a dual role in skin by inducing the innate immune response, but predisposing to systemic immunosuppression and genomic mutations [1, 92, 97]. It is not known, what is the feasible level of UV exposure and to what extent lncRNAs can mediate the balance between skin homeostasis and carcinogenesis.

## LncRNAs in keratinocyte carcinomas

The UV-induced alteration of lncRNA expression in epidermal cells suggests that some of these lncRNAs exert a protective role against carcinogenesis by triggering UV-induced early stress response [98–105] (Table 1). On the other hand, some of them may play a role at the early stage of epidermal carcinogenesis and loss of some differentiation-associated lncRNAs may serve as markers for tumor initiation. In keratinocyte carcinomas, particularly in cSCCs, several lncRNAs are differentially expressed as compared to normal skin or keratinocytes, suggesting a role for them in cSCC progression [109, 110]. Some of the deregulated lncRNAs may function in signaling pathways, which are already mutationally activated or suppressed in cSCC. On the other hand, it is likely that some of these lncRNAs are targeted by UV-induced mutations or by genomic alterations within the lncRNA gene itself, as has been observed in several cancer cell lines [111, 112]. As none of the BCC-associated lncRNAs have been functionally characterized so far, we will focus on lncRNAs implicated in cSCC (Table 1).

**Table 1:** Long non-coding RNAs with a potential role in cSCC or BCC development

LncRNA	Expression	Function	References
*TINCR*	Downregulated in cSCC	Promotes human epidermal differentiation by stabilization of mRNAs coding for differentiation specific genes	[68]
*SMRT-2*	Downregulated in cSCC	Induced during keratinocyte differentiation. Knockdown in human organotypic skin downregulates several differentiation specific genes, including ZNF750 and KLF4	[74]
*LINC00520*	Downregulated in cSCC	Inhibits cSCC progression by downregulating expression of EGFR and its downstream targets, *e.g.* PI3K, AKT, and VEGF	[123]
*PICSAR*	Upregulated in cSCC	Promotes cSCC progression by activating ERK1/2 by downregulating DUSP6. Decreases cSCC cell adhesion and increases cSCC cell migration by downregulating integrin expression	[110, 118]
*PRECSIT*	Upregulated in cSCC	Promotes cSCC cell invasion through STAT3-mediated upregulation of production of MMP-13, MMP-3, MMP-1, and MMP-10	[129]
*LINC00319*	Upregulated in cSCC	Increases cSCC cell growth, migration, and invasion. Suppresses apoptosis by upregulating cyclin-dependent kinase 3 via miR‐1207‐5p decoy	[130]
*LINC01048*	Upregulated in cSCC	Interacts with TAF15 transcription factor to induce YAP1 transcription and tumorigenic function via Hippo signaling pathway	[126]
*MALAT1*	Upregulated in cSCC	Positively regulates EGFR protein expression via c-MYC and KTN1	[119]
*lincRNA-p21*	Induced in mouse and human keratinocytes by UVB	Tumor suppressive role by triggering UVB-induced apoptotic death	[105]
*AK144841*	Induced in mouse DMBA/TPA-induced cSCC	Downregulates several anticancer and cell differentiation genes in mouse	[79]
*H19,Hottip, Nespas, mHOTAIR, MALAT1, SRA*	Upregulated in vitamin D receptor (VDR) deleted mouse keratinocytes and epidermis	Potential oncogenes in skin cancer progression	[108]
*Kcnq1ot1, lincRNA‐p21, Foxn2‐as, Gtl2‐as, H19‐as*	Inhibited in VDR-deleted mouse keratinocytes and epidermis	Potential tumor suppressors in skin cancer formation	[108]
*H19, CASC15, SPRY4-IT*	Upregulated in BCC	Potential oncogenes in BCC	[131]

Aberrant activation of the ERK1/2 MAPK pathway is one of the central drivers in the molecular pathogenesis of cSCC [113–115]. ERK1/2 pathway is activated by UVA radiation [116]. Moreover, mutational activation of epidermal growth factor receptor (EGFR) results in sustained activation of the RAS-RAF-MEK-ERK signaling pathway and promotes cutaneous carcinogenesis [117].

### *PICSAR* plays a tumorigenic role in cSCC

**Fig. 3:**
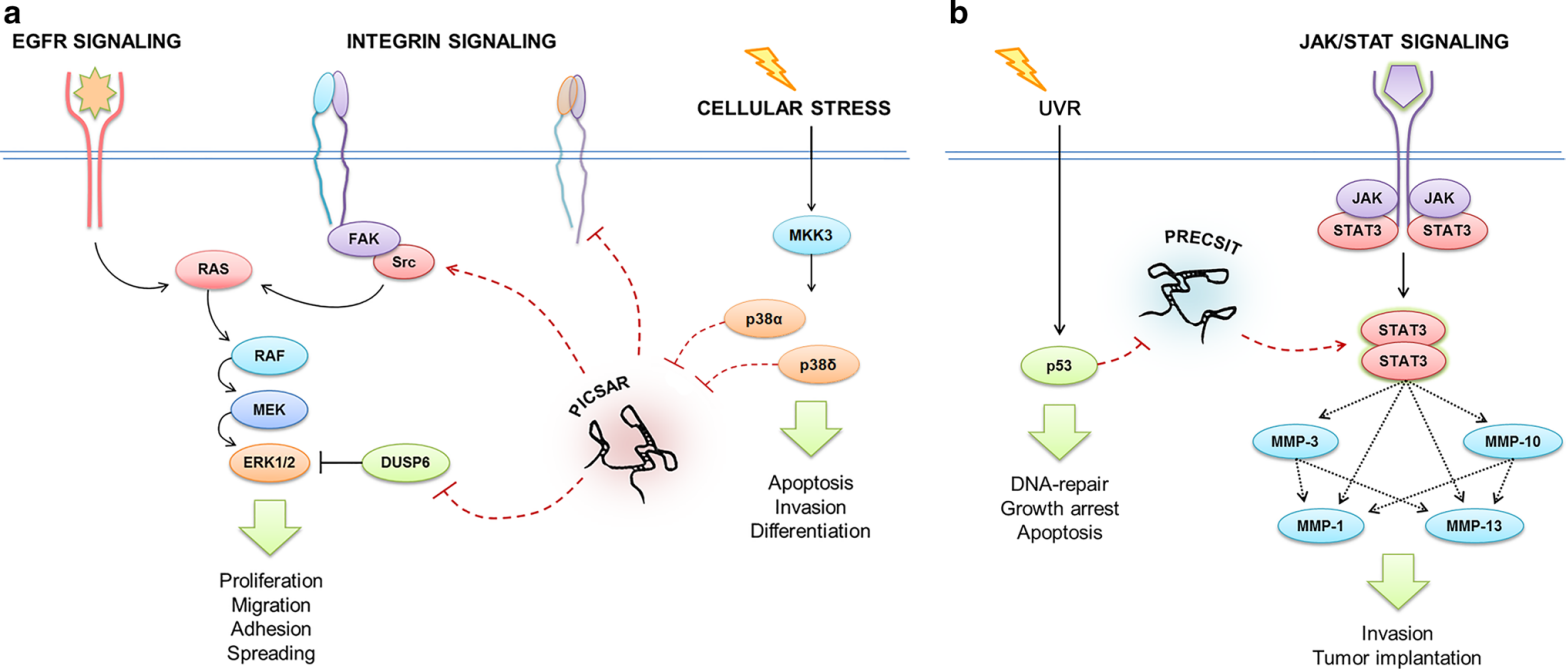
Proposed molecular functions for lncRNAs *PICSAR* and *PRECSIT* in cSCC. **a** Expression of *PICSAR* is suppressed by the p38 signaling pathway. *PICSAR* promotes activity of ERK1/2 and cell proliferation by inhibiting expression of dual-specificity phosphatase DUSP6 in cSCC cells. In addition, *PICSAR* modulates cSCC cell adhesion and migration by regulating integrin expression on the cell surface. **b** Expression of *PRECSIT* is suppressed by functional p53 signaling, and elevated *PRECSIT* expression in response to p53 inactivation contributes to STAT3 activation, which in turn upregulates matrix metalloproteinases MMP-13, MMP-3, MMP-1, and MMP-10 in the MMP cluster in 11q22.3 and this way promotes proteolytic remodeling of extracellular matrix and basement membrane, and cSCC cell invasion

p38-inhibited cutaneous squamous cell carcinoma-associated lincRNA (*PICSAR*) represents the earliest evidence of a functionally characterized lncRNA in cSCC [110]. The expression of *PICSAR* is upregulated in cSCC tumor cells in culture and in vivo compared to normal human epidermal keratinocytes (NHEKs) and normal skin [110]. Elevated expression of *PICSAR* was also noted in vivo in actinic keratosis and cSCC in situ, suggesting a role for *PICSAR* at the early stage of epidermal carcinogenesis [110]. Silencing of *PICSAR* expression potently suppresses growth of human cSCC xenografts [110]. Interestingly, *PICSAR* serves as a regulatory link between p38 and ERK1/2 mitogen-activated protein kinase (MAPK) pathways (Fig. 3a). Inhibition of p38 activity induces *PICSAR* expression and *PICSAR* promotes cSCC cell proliferation by promoting ERK1/2 activity via downregulation of dual specificity phosphatase DUSP6 [110]. In addition, *PICSAR* potently regulates cell adhesion and migration by regulating integrin expression [118], and may this way contribute to cSCC progression and invasion (Fig. 3a).

### *MALAT1* and *LINC00520* play opposite roles in cSCC

*MALAT1* is a lncRNA, which has been reported to be deregulated in different types of cancer [85]. Elevated expression of *MALAT1* was recently reported in cSCC tumors and it was shown that the expression in cSCC cells is induced by UVB [119]. *MALAT1* promotes proliferation, migration, and invasion of cSCC cells and growth of cSCC tumors in vivo and suppresses apoptosis of cSCC cells [119]. Mechanistically, *MALAT1* interacts with c-Myc to activate transcription of kinectin 1 (*KTN1*) gene, which is one of the top downregulated genes after *MALAT1* depletion. Knockdown of *MALAT1* also results in decreased level of EGFR protein, but not EGFR mRNA [119]. These results suggest that *MALAT1* contributes to cSCC pathogenesis by upregulating EGFR protein levels via c-Myc and *KTN1* [119].

Marked expression of lncRNA *AK144841* has been noted in chemically (DMBA/TPA) induced mouse cSCCs compared to healthy skin [79]. The histology and the genomic background of these tumors are very similar to human cSCCs [120]. Sustained activation of HRAS, which is caused by highly carcinogenic DMBA, results in marked induction of EGFR and its ligands in cSCC mouse model [121, 122]. In this regard, induction of *AK144841* in murine cSCC may be related to EGFR activation. A potential human ortholog with homology to *AK144841* has been shown to be expressed at high level in cSCC cell lines compared to NHEKs suggesting, that it may be involved in human cSCC progression [79].

Downregulation of *LINC00520* has been noted in A431 cSCC cell line, compared to NHEKs, and overexpression of *LINC00520* in A431 cells results in suppression of tumor growth and lymph node metastasis [123]. A431 cells express high levels of EGFR [124]. Reduced expression of EGFR and its downstream targets, PI3K, AKT, VEGF, MMP-2, and MMP-9 was noted in A431 cells overexpressing *LINC00520*, whereas an opposite effect was noted after *LINC00520* depletion [123]. Altogether, these results suggest that *LINC00520* plays a tumor suppressive role in cSCC by targeting EGFR [123].

### *TINCR* and *SMRT-2* are potential tumor suppressors in cSCC

Poor differentiation of cSCC is associated with risk for metastasis and poor prognosis [77, 78]. *TINCR* and *SMRT-2* both promote differentiation of keratinocytes and may this way serve in a protective role in keratinocyte carcinogenesis [68, 74]. Accordingly, decreased expression of *TINCR* and *SMRT-2* has been noted in human cSCCs [68, 74], and a notable decrease in *TINCR* expression has been reported in DMBA/TPA-induced murine cSCC tumors compared to normal skin [79]. In addition, marked suppression of *SMRT-2* expression has been noted in Ras-driven human organotypic epidermal neoplasia [74]. Together, these two lncRNAs may function as potential tumor suppressors in cSCC. In this context, it is interesting that ZNF750 which upregulates the expression of *TINCR* in keratinocytes, was recently shown to exert a tumor-suppressive role in SCCs of head and neck, lung, cervix, and skin [76].

### *LINC01048* and Hippo pathway in keratinocyte carcinoma

Hippo pathway is a well-conserved signaling pathway, which is important in skin development, cutaneous homeostasis and tissue regeneration, and aberrant Hippo signaling has been noted in non-melanoma skin cancers [125]. Recently, upregulation of a previously unknown lncRNA, *LINC01048*, was reported in cSCC associated with lower overall survival of cSCC patients [126]. *LINC01048* promotes cSCC cell growth via the Hippo pathway [126]. Depletion of *LINC01048* regulates the levels of the downstream effectors of the Hippo signaling, including yes-associated protein 1 (YAP1) and transcriptional co-activator with PDZ-binding motif (TAZ). Mechanistically, *LINC01048* interacts with transcription factor TAF15 to promote transcription of *YAP1* gene [126]. Accordingly, *YAP1* and *TAZ* function as oncogenes in many cancers, including BCC and cSCC [127, 128]. Together these results provide interesting new evidence for the role of *LINC01048*/TAF15/YAP1-axis in cSCC progression.

### *PRECSIT* and *LINC00319* regulate invasion of cSCC

p53-regulated carcinoma-associated STAT3-activating long intergenic non-protein coding transcript (*PRECSIT*) is a recently identified lncRNA with elevated expression in cSCC [129]. *PRECSIT* is a nuclear-enriched lncRNA downregulated by p53 signaling, and a high level of *PRECSIT* expression is associated with the absence of functional p53 in cSCC tumor cells in vivo [129]. Depletion of *PRECSIT* inhibits cSCC cell invasion by downregulating STAT3 expression and activation, and production of matrix metalloproteinases (MMPs), MMP-13, MMP-3, MMP-1, and MMP-10 [129], suggesting a tumor-promoting function for *PRECSIT* (Fig. 3b). These results provide interesting new evidence that p53/PRECSIT/STAT3 axis regulates the expression of invasion proteinases in the MMP gene cluster in 11q22.3: MMP-13/MMP-3/MMP-1/MMP-10.

*LINC00319* is a recently identified lncRNA with elevated expression in cSCC shown to correlate with larger tumor size and lymphovascular invasion of cSCC [130]. *LINC00319* promotes cSCC cell migration and invasion, and upregulates expression of MMP-2, MMP-9, and markers for epithelial–mesenchymal transition, E-cadherin, and vimentin [130]. *PRECSIT* regulates the invasion of cSCC cells specifically without affecting cell growth [129], whereas *LINC00319* has an anti-apoptotic function and promotes cSCC cell proliferation via miRNA-mediated mechanism [130].

## Concluding remarks

The role of lncRNAs in epidermal biology is slowly emerging. The recent findings summarized here elucidate the functional role of lncRNAs in physiological conditions and keratinocyte cancer development, specifically in cSCC (Fig. 2, Table 1). It is noteworthy, that none of the BCC-associated lncRNAs have been functionally characterized yet. Moreover, considering UVR as a common nominator for the development of cSCC and BCC, it remains unclear whether they share the same UV-regulated lncRNAs. These cancers have distinct mutational background and different oncogenic signaling pathways. Therefore, it is likely that there are also specific lncRNAs which, by function, are associated with either cSCC or BCC development by co-operating with various signaling molecules to mediate the expression of tumor promoting or tumor suppressing genes. LncRNAs present great potential in developing new diagnostic and therapeutic approaches. Along with conventional molecular markers, distinct lncRNA expression signature may provide better diagnostic accuracy of the disease. Moreover, therapeutic targeting of tumorigenic lncRNAs may enhance the efficacy of cancer therapy.
